# Identification of Two Novel Mutations of *ABCD1* Gene in Pedigrees with X-Linked Adrenoleukodystrophy and Review of the Literature

**DOI:** 10.1155/2022/5479781

**Published:** 2022-02-07

**Authors:** Bingzi Dong, Wenshan Lv, Lili Xu, Yuhang Zhao, Xiaofang Sun, Zhongchao Wang, Bingfei Cheng, Zhengju Fu, Yangang Wang

**Affiliations:** Department of Endocrinology and Metabolism, The Affiliated Hospital of Qingdao University, Qingdao 266003, China

## Abstract

**Background:**

X-linked adrenoleukodystrophy (ALD) is an inherited peroxisomal metabolism disorder, resulting from the loss-of-function mutation of ATP-binding cassette protein subfamily D1 (*ABCD1*) gene. The dysfunction of ALD protein, a peroxisomal ATP-binding cassette transporter, results in the excessive saturated very long-chain fatty acids (VLCFAs) accumulation in organs including the brain, spine, and adrenal cortex. X-ALD is characterized as the childhood, adolescent, adult cerebral ALD, adrenomyeloneuropathy (AMN), adrenal insufficiency, and asymptomatic phenotypes, exhibiting a high variety of clinical neurological manifestations with or without adrenocortical insufficiency.

**Results:**

In this study, we reported two cases of X-ALD, which were first diagnosed as adrenal insufficiency (Addison's disease) and treated with adrenocortical supplement. However, both of the cases progressed as neurological symptoms and signs after decades. Elevated VLCFAs level, brain MRI scan, and genetic analysis confirmed final diagnosis. In addition, we identified two novel mutations of *ABCD1* gene, NM_000033.3 (ABCD1): c.874_876delGAG (p.Glu292del) and NM_000033.3 (ABCD1): c.96_97delCT (p.Tyr33Profs*∗*161), in exon 1 of *ABCD1* gene. Sanger sequencing confirmed that the proband's mother of the first case was heterozygous carrying the same variant. Adrenal insufficiency-only type is very rare; however, it may be the starting performance of X-ALD. In addition, we summarized reported mutation sites and clinical manifestations to investigate the correlationship of phenotype-genotype of X-ALD.

**Conclusions:**

The early warning manifestations should be noticed, and the probability of X-ALD should be considered. This report could be beneficial for the early diagnosis and genetic counseling for patients with X-ALD.

## 1. Background

X-linked adrenoleukodystrophy (X-ALD) (OMIM#300100) is a X-linked inherited peroxisome metabolic neurodegenerative disorder, resulted from the defects in ATP-binding cassette protein subfamily D1 (*ABCD1*) gene. *ABCD1* gene locates in chromosome Xq28, contains 10 exons, and codes the adrenoleukodystrophy protein (ALDP), a member of the ABC transporter superfamily. ALDP expresses in the peroxisome membrane, containing 745 amino acids. It plays a key role in VLCFAs transporting into peroxisome for *β*-oxidation and degradation [1]. The defective ALDP results in the impaired degradation of saturated very long-chain fatty acids (VLCFAs). Excessive VLCFAs accumulate in plasma and tissues including adrenal cortex, cerebral white matter, or spinal cord [2] to exhibit clinical manifestations. Till now, there are more than three thousand variants reported (X-ALD mutation database, http://www.x-ald.nl/) [3].

Based on the age of onset and the extent of organs affected, X-ALD is classified into seven clinical subtypes: childhood cerebral, adolescent cerebral, adult cerebral, adrenomyeloneuropathic (AMN), cerebellar, adrenal insufficiency-only (Addison's disease), and asymptomatic or presymptomatic types. X-ALD exhibits a high variety of clinical characteristics [4]. Childhood cerebral ALD (CCALD) and adult-onset AMN are the two most common phenotypes [5]. The clinical symptoms include progressive dysfunction of central and peripheral nervous systems, such asparalysis, behavioral disturbance, abnormalities of sphincter control, sensory loss, or paresthesia, with or without adrenal insufficiency. Brain MRI shows a typical pattern of symmetrical enhanced T2WI signal in the bilateral white matter around the lateral ventricle, cerebellum, and the genu of the corpus callosum [6]. Significantly elevated level of VLCFAs is the important diagnostic clue for the individuals with ALD. There are three parameters of VLCFAs to analyze the concentration of C26 : 0, the ratio of C24 : 0/C22 : 0, and the ratio of C26 : 0/C22 : 0 [5]. Final diagnosis relays on the genetic analysis to detect *ABCD1* mutation.

Adrenal insufficiency-only type is rare, especially in females less than 1% [7, 8]. Some individuals were diagnosed as Addison's disease without symptoms of cerebral or myelopathy ALD for decades. However, the early onset Addison's disease may gradually progress to exhibit neuropsychiatric symptoms, showing a time-dependent follow-up [9].

In this study, we reported two pedigrees of X-ALD patients first diagnosed as Addison's disease for decades and gradually exhibited variable clinical neuropsychiatric manifestations in their late twenty's and showed rapid progression and poor prognosis. In addition, we identified two novel mutations of *ABCD1* gene, NM_000033.3 (ABCD1): c.874_876delGAG (p.Glu292del) and NM_000033.3 (ABCD1): c.96_97delCT (p.Tyr33Profs*∗*161), in the two pedigrees of X-ALD. Further study of genotype-phenotype correlation analysis is needed to provide deeper insights into X-ALD.

## 2. Methods and Materials

### 2.1. Patients' Recruitment

The diagnosis of X-ALD depends on clinical symptoms, biochemical parameters, neurological imaging, and genetic analysis. This study was approved by the Ethics Committee of the Affiliated Hospital of Qingdao University.

### 2.2. Genetic Analysis and Prediction of Pathogenicity

Genomic DNA was extracted from peripheral blood sample using the QIAamp Blood DNA Mini Kit (QIAGEN, USA) according to the manufacturer's protocol. After amplification using 2X polymerase chain reactions (PCR) MasterMix polymerase (Tiangen, China) by ABI9700 PCR (Life technology, USA), the products were captured and purified with Panel probe (illumine Inc. USA) and then directly sequenced on the ABI 3500 automated DNA sequencer (Life Technology, USA). Mutations of *ABCD1* were detected using next generation sequencing (NGS) and subsequently confirmed using Sanger sequencing. The databases including ESP6500, 1000 Genomes, and dbSNP and UniProt were used for elimination of polymorphisms. Prediction of mutations was performed with PROVEAN (Protein Variation Effect Analyzer, http://provean.jcvi.org/) and Mutation Taster (http://www.mutationtaster.org) to predict the possible impacts of amino acid substitution on the structure and function. The PROVEAN score represented the normalized probability that the amino acid change is deleterious. The cutoff of PROVEAN score was −2.5. The PROVEAN score <−2.5 was predicted to be deleterious. In addition, the following forty genes associated with congenital adrenal hypoplasia or glucocorticoid deficiency were investigated for Case 2, including *MC2R*, *MRAP*, *AAAS*, *AIP*, *PRKAR1A*, *PDE11A*, *PDE8B*, *PRKACA*, *MEN1*, *RET*, *GNAS*, *ARMC5*, *NF1*, *NR3C1*, *LMNA*, *SOX3*, *PCSK1*, *PROP1*, *LHX3*, *HSD11B1*, *CYP21A2*, *CYP17A1*, *CYP11B1*, *HSD3B2*, *STAR*, *MCM4*, *TBX9*, *POR*, *POMC*, *AIRE*, *NR5A1*, and so on.

### 2.3. Three-Dimensional Structure Prediction of Protein and the Potential Dysfunction

We identified the alteration of the *ABCD1*-encoded ALD protein structure and potential dysfunction using the SWISS-MODEL workspace (http://swiss-model.expasy.org).

### 2.4. Case Presentations and Biochemistry Results

**Case 1 .**

**Figure 1:**
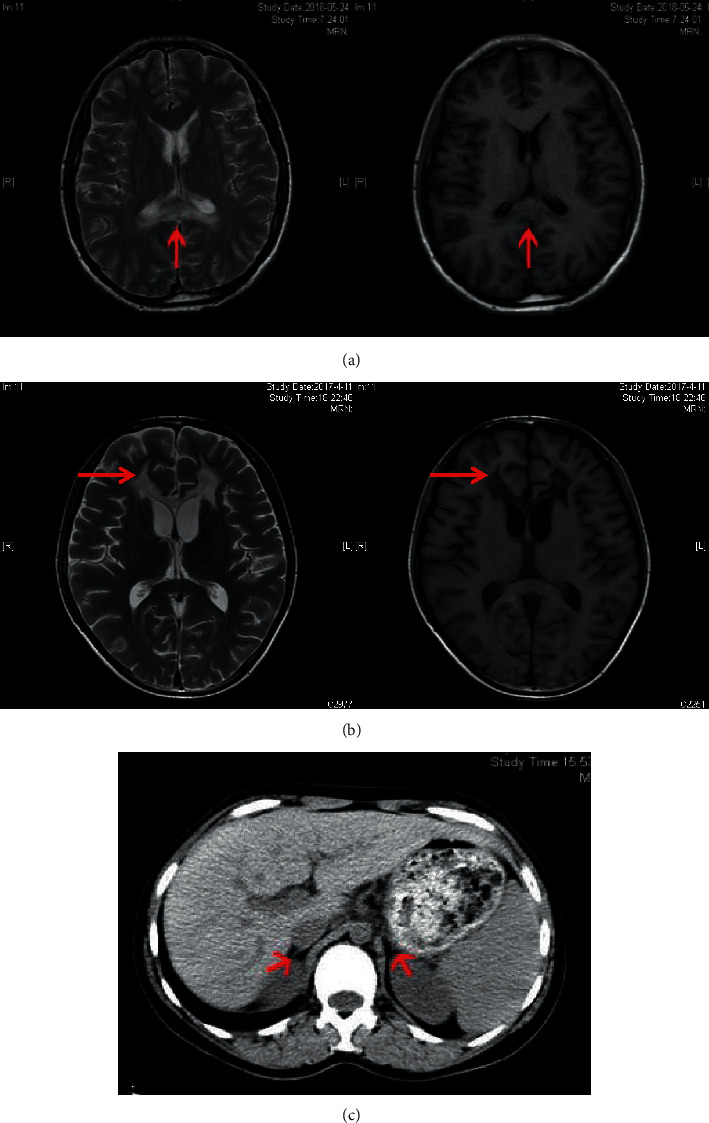
Brain magnetic resonance imaging (MRI) scan of patients. (a) Symmetric bands with high T2 low T1 abnormal signal in bilateral genu of the corpus callosum detected in case one. (b) MRI scan of case two revealed long T1 and long T2 signals in white matter of bilateral frontal lobes, around the anterior of lateral ventricle, and splenium of the corpus callosum. (c) Computed tomography (CT) scan of the adrenal gland of case one to show bilateral adrenal atrophy. Red arrow shows lesions.

**Figure 2:**
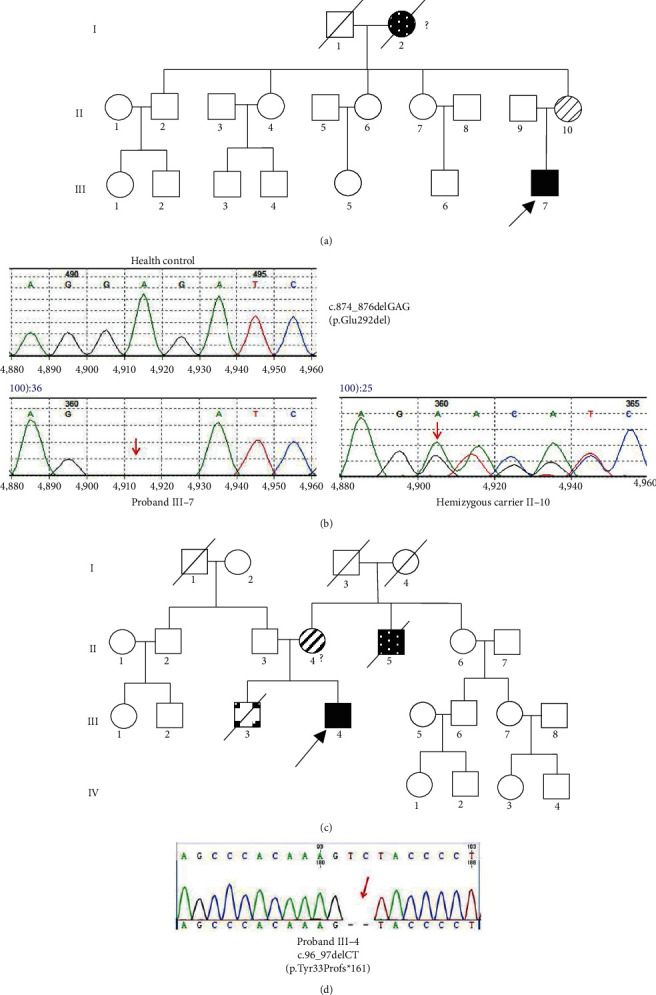
Pedigree diagrams and genetic analysis of two cases. (a), (c) Pedigree diagrams of the case one and case two with X-linked ALD, respectively. Empty square and circle show healthy male and female. Dark square represents patients with X-ALD. Diagonal stripes show heterozygous carrier. Arrow represents hemizygous proband. (b) Genetic analysis revealing novel mutation of c.874_876delGAG (p.Glu292del) of *ABCD1* gene in patient one (proband III-7) and his mother (II-10). (d) Novel mutation c.96_97delCT (p.Tyr33Profs^*∗*^161) detected in patient two (proband III-4).

A 27-year-old male was presented in hospital with weakness, fatigue, poor academic performance, exhibiting skin, and mucous membrane pigmentation, especially in areolae, joints, and axilla for two decades. The cortisol at 8 am was reduced 3.58 nmol/L (range 171–536 nmol/L), and adrenocorticotropic hormone (ACTH) was increased 499.30 pg/mL (range 7.2–63.6 pg/mL). After diagnosed as primary adrenal insufficiency (Addison's disease), he was administrated with glucocorticoid replacement (hydrocortisone 20 mg per day) for one year, and the skin pigmentation and weakness improved. In recent half a year, the patient acted as mild cognitive impairment, slurred speech, ataxia, and rapidly progressed as walking instability, incontinence, and paroxysmal blurring vision. Physical examination detected scanty scalp hair, V-grade muscle strength, hypomyotonia, tendon hyperreflexia (+++), and positive Babinski's sign and Gordon's sign. The light and deep reflexes were normal. The heel-to-knee test, alternating movement test, and Romberg's test were negative. The respiratory, cardiovascular, and abdominal examinations were unremarkable. Brain MRI showed symmetrical distribution of hyperintensity long T1 and long T2 signal in bilateral splenium of corpus callosum and widened fissures and sulci (Figure 1(a)). Serum VLCFA levels were significantly increased in the proband and also elevated in the proband's mother (Table 1, Figure 2(a), II-10). Until now, his mother did not show manifestations of cerebral disorders, myeleterosis, or adrenal insufficiency. His maternal grandmother (Figure 2(a), I-2) died at her 55-year-old due to encephalopathy. However, family members were unable to provide valid information, and no blood samples could be obtained for testing. Other siblings of his mother did not show neuropsychiatric manifestations. Genetic analysis revealed variants c.874_876delGAG of *ABCD1* gene in exon 1. According to the clinical manifestations and genetic analysis, a pedigree of X-ALD was constructed. The patient was administrated with hydrocortisone 20 mg per day, mecobalamine, and compound vitamin B. During the follow-up, the proband showed rapidly progressed neuropsychiatric manifestations including blurred vision, instability of gait, and incontinence within six months.

**Case 2 .**

A 31-year-old male was presented with adrenal crisis, exhibiting severe fatigue, anorexia, nausea, and vomiting five years ago. The patient had significantly generalized skin pigmentation especially in the gingiva, areolae, and creases of the hands. Laboratory examination revealed severe hyponatremia (serum sodium 108–125 mmol/L, normal range 145–155 mmol/L), markedly decreased cortisol level (24.83-45.40-35.13 nmol/L) and increased ACTH level (1522-1071-1750 pg/mL) at 8 am-4 pm-0 am, respectively. The adrenal CT scan showed bilateral adrenal atrophy. At first, the patient was diagnosed as adrenal insufficiency, without exhibiting central or peripheral neuropsychiatric symptoms. After therapy with hydrocortisone (20 mg at 8 am, −10 mg at 4 pm), symptoms of fatigues and vomiting improved. The patient showed incorrigible severe hyponatremia, only corrected into normal by treated combination with fludrocortisone (0.05 mg per day). The serum sodium levels were maintained in normal range for five years. However, in recent eight months, the patient showed significantly aggravated walking instability, cognitive dysfunction, intellectual disabilities, emotional disturbance, memory loss, and incontinence. Neurological physical examination revealed positive Babinski's sign and Hoffman's sign, hyperactive tendon reflexes, and abnormal heel-to-knee-to-toe test. He spoke unclearly with dysarthria, while vision and hearing were normal. Brain MRI revealed long T1 and long T2 signals in white matter of bilateral frontal lobes and around the anterior of lateral ventricle and corpus callosum (Figure 1(b)). The patient was sick and showed poor learning ability during childhood. His brother (Figure 2(c), III-3) also showed sickness in childhood and died at his 8-year-old. His maternal uncle (Figure 2(c), IL-5) was weak and fatigued in his youth and showed disturbance of consciousness from 25-year-old and died at 28-year-old. None of the other family members exhibited neuropsychiatric symptoms and signs of X-ALD. To confirm the diagnosis, genetic sequencing of the proband detected deletion mutation c.96_97delCT in *ABCD1* gene. Thus, the pedigree of X-ALD was suspected. During the follow-up, the patient was treated with hydrocortisone and fludrocortisone, and serum sodium level was maintained in normal range. However, he showed rapidly progressed symptoms of incontinence and consciousness dysfunction.

### 2.5. Genetic Analysis and Prediction of Pathogenicity

Genetic sequencing of *ABCD1* gene was performed on the probands of two cases and the familial relatives of the proband in case one. In case one, genetic sequencing detected heterozygous mutation of NM_000033.3 (ABCD1): c.874_876delGAG (p.Glu292del) in *ABCD1* gene exon 1 (Figures 2(a) and 2(b)), leading to the deletion of translation product glutamate and accumulation of VLCFAs (Table 1). Sanger sequencing of *ABCD1* gene was performed on his mother and confirmed the heterozygous point mutation for the same variant. In case two, gene sequencing showed a deletion mutation c.96_97delCT in *ABCD1* gene (Figures 2(c) and 2(d)), leading to the frameshift and premature transcription termination of amino acid p.Tyr33Profs*∗*161. His immediate family members (parents and his sister) refused to perform genetic sequencing.

The frequency of those variants was examined in the reference database. Both novel mutations were not detected in the database including ESP6500, 1000 Genomes, or dbSNP (Table 2). The potential pathogenicity of both mutations was investigated using prediction bioinformatics, suggesting both mutations were possible to be disease causing (Table 2). The pathogenicity evaluation was analyzed according to the criteria of American College of Medical Genetics (ACMG) guideline and given in Table 2.

### 2.6. Three-Dimensional Structure Prediction of Protein and Potential Dysfunction

**Figure 3:**
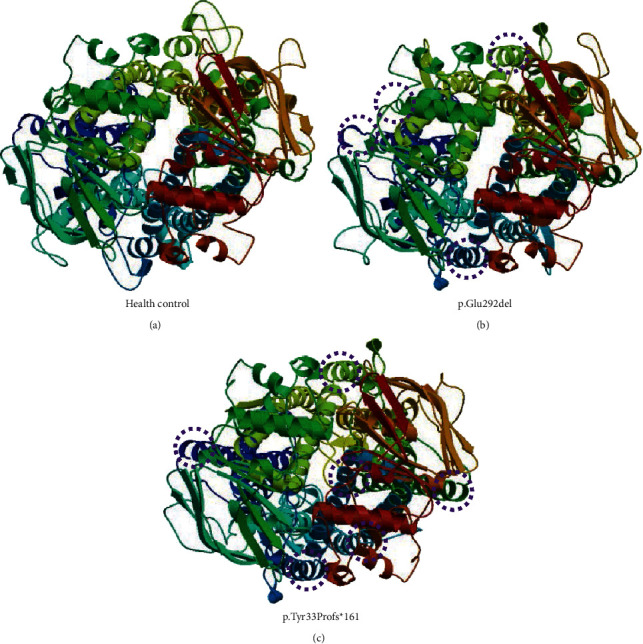
Three-dimensional structure prediction of adrenoleukodystrophy (ALD) protein. (a) The modeled structure of health control. The visible alteration of ALD protein structure induced by mutations (b) c.874_876delGAG (p.Glu292del) and (c) c.96_97delCT (p.Tyr33Profs*∗*161) are indicated in circles. It indicates that those mutations might lead to alteration of the protein structure and potential dysfunction.

The *ABCD1* gene-encoded ALD protein contains transmembrane domains and three segments on the peroxisome side. We identified the predicted alteration of the ALD protein structure and its potential dysfunction induced by mutations NM_000033.3 (ABCD1): c.874_876delGAG (p.Glu292del) and NM_000033.3 (ABCD1): c.96_97delCT (p.Tyr33Profs*∗*161) in silico (Figure 3). In both of the mutations, p.Glu292del and p.Tyr33Profs*∗*161, the amino acid changes in ALD protein due to those deletion mutations of *ABCD1* gene caused more than three visible differences in protein structure, might affect function, leading to the impaired degradation of VLCFAs and inflammation environment.

## 3. Discussion

X-ALD is a demyelinating and neurodegenerative disorder due to the loss-of-function mutation of *ABCD1* gene. *ABCD1* gene codes the peroxisome transporter protein ALDP, which plays an important role in regulating peroxisome oxidation and degradation of VLCFAs [5]. The dysfunction of ALDP, a fatty acid transporter, results in the impaired VLCFA oxidation and excessive VLCFAs accumulation in tissues [5, 10]. Excessive VLCFAs in cytoplasm lead to mitochondrion dysfunction and ER stress and finally caused cell death. In addition, accumulated VLCFAs arouse inflammation, resulting in demyelinating and neurodegeneration.

In this study, we reported two Chinese X-ALD pedigrees. Both of the probands were diagnosed as adrenal insufficiency at beginning without neuropsychiatric symptoms for decades, but developed into neurological disorders at their late 20s and showed rapid progression and poor prognosis. Two novel mutations in exon 1 of *ABCD1* gene were detected and suggested to be associated with X-ALD.

According to the previous reports, the majority is cerebral ALD among those seven types of ALD [11]. CCALD is early onset with a feature of rapidly progressed neuroinflammatory of cerebral demyelination. Neurological symptoms of AMN commonly occur from the ages of 20s, with manifests as a chronic progressive paraparesis, accompanied with sensory and motor disturbances [12]. Due to the variable clinical manifestations, some are difficult to make the diagnose at the early stage [13]. Adrenocortical insufficiency was reported as the first clinical manifestation of 38% X-ALD, greater than the group of spinal cord 25% and cerebral disorders 14.5% [9]. To be noticed, some of those male patients diagnosed as adrenal insufficiency may develop with cerebral or myeloneuropathy ultimately and show rapid progressive neurological symptoms and poor prognosis. On the contrast, female ALD patients rarely show adrenocortical insufficiency or cerebral ALD. Most of female patients suffer from lately onset myelopathy [10, 14]. We reported two cases; both were first diagnosed as Addison's disease, and then, the therapy focused on the adrenocortical replacement. However, after the neurological symptoms and signs become apparent, both of the cases were rapidly progressed. The therapy including fatty acid restriction or neurotrophic treatment did not show the therapeutic effect. Therefore, at diagnosis of congenital adrenal insufficiency, the probability of ALD should be fully considered, especially for male patients [8, 15]. To make the early diagnosis, family history, MRI scan of brain or spinal cord, VLCFAs measurement, and genetic screening may be helpful for those patients with subtle early neurological manifestations.

We identified two novel point mutations in exon 1 of *ABCD1* gene in the pedigree of first case NM_000033.3 (ABCD1): c.874_876delGAG (p.Glu292del) and in second case NM_000033.3 (ABCD1): c.96_97delCT (p.Tyr33Profs^*∗*^161). The genetic sequencing demonstrated nucleotides deletion, resulting in those frameshift mutations. Based on the clinical symptoms and positive neurologic signs, family history, imaging, biochemical characteristics, and the results of pathogenicity prediction using bioinformatics tools, we suggest those mutations as pathogenic variants. However, the biopsy of the brain, spinal cord, and adrenal gland was not done. Based on the elevated plasma VLCFAs, the deleterious change of ALDP impaired the cellular beta-oxidation of fatty acid [16].

**Figure 4:**
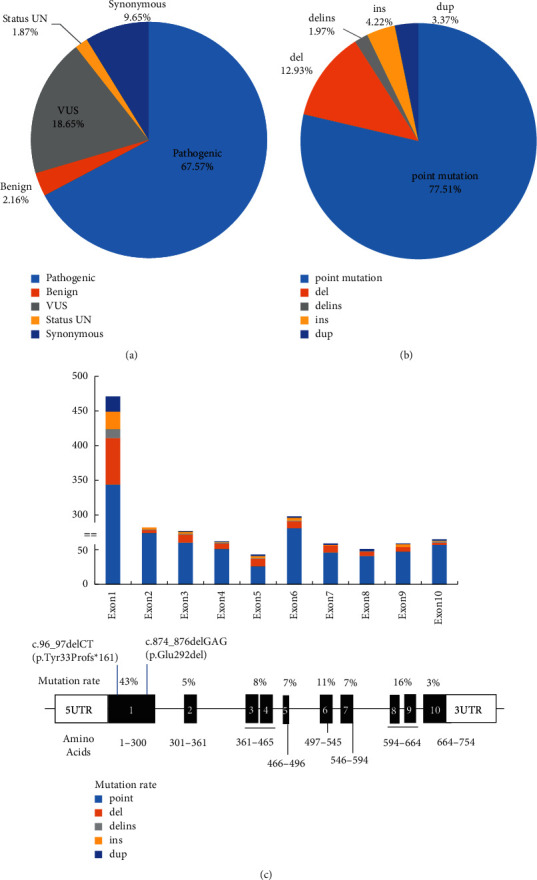
*ABCD1* gene reported from the ALD mutation database. (a) Mutation sites types of variants of *ABCD1* gene among reported nonrecurrent and other mutation sites, including pathogenic, synonymous, benign, variants of undetermined significance (VUS), and screening of newborns state of unknown (UN). (b) The content of *ABCD1* mutations, including points mutations, deletion, del-insertion, insertion, and duplication (http://www.x-ald.nl/). (c) The distribution of mutations, including point mutation, deletion, del-insertion, insertion, and duplication. Most of the mutations harboring in exon 1. The lower panel shows the mutation rate in each exon. Bold shows the location of two novel mutations indicated in this study.

**Figure 5:**
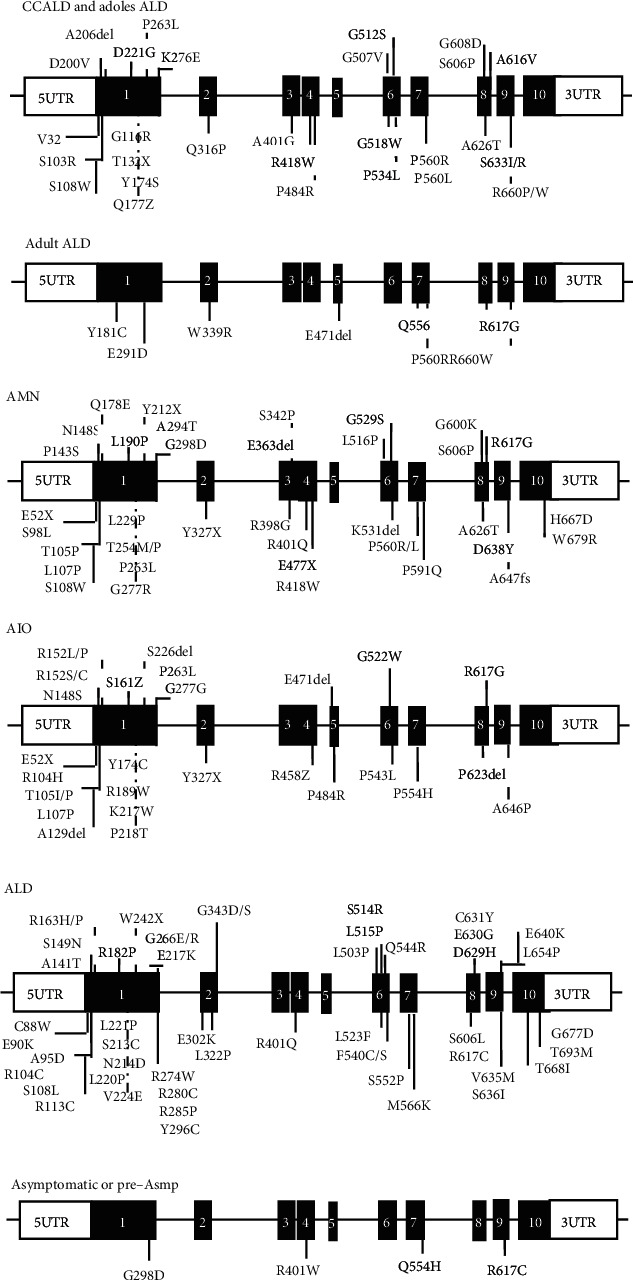
Summary of the reported mutation sites and clinical manifestations from the database of UniProt (https://www.uniprot.org/uniprot/) and reported references [22–35]. The clinical types include childhood and adolescent cerebral ALD (CCALD and adoles ALD), adult ALD, AMN (adrenomyeloneuropathy), adrenal insufficiency-only (AIO), complicated ALD which cannot be classified, and asymptomatic or presymptomatic types.

There are more than three thousand mutations reported in the ALD database (http://www.x-ald.nl/). Among them, over 900 variant sites are nonrecurrent unique. Most of them are pathogenic mutations. Among more than thousand mutation sites (combination of 913 nonrecurrent mutations, 120 synonymous variants, and 247 variants of unknown significance), 67.57% of them are pathogenic mutations, 9.65% are synonymous, 2.16% are benign, 18.65% are VUS (variant of undetermined significance), and 1.87% are status unknown mutations detected during the screening of newborns (Figure 4(a)). More than 77.5% variant sites are point mutations; other types include 12.93% deletion, 1.97% del-insertion, 4.22% insertion, and 3.37% duplication (Figure 4(b)). Most of the mutations locate in the exon (92.94%) and others in the intron (6.18%), 5'UTR (0.61%), and 3'UTR (0.26%), respectively [4]. In the database, nearly half of the mutations were reported anchoring in exon 1 (Figure 4(c)), which encodes the transmembrane domain (TMD) of the protein, contains ligand-specific binding sites, and plays an important role in the localization of ALDP [17]. Another clustering of mutations occurs in exon 6, which encodes the ATP-binding domain of ALDP [18]. ALDP locates in the peroxisome membrane to transport VLCFAs from cytosol into the peroxisome for degradation. Pathogenic *ABCD1* mutations may induce defective stability of ALDP transmembrane structure region and ATPase activity to affect the localization of VLCFAs to the peroxisome correctly. As a result, VLCFAs cannot be transported into peroxisomes for *β*-oxidation and then accumulate mainly in the white matter, neuron axons of the central nervous system, and adrenal cortex. Excess VLCFAs accumulation leads to destructive progression by triggering oxidative stress and mitochondrial dysfunction, chronic inflammation, lipid-induced neuron apoptosis, and degenerative changes in the nervous system, and finally lead to neurodegeneration and adrenal insufficiency [3, 19]. Though some variations may not affect protein translation or function, they still represent polymorphisms. The genotype-phenotype correlationship has not been identified clearly [20, 21]. To further investigate the relationship between genotype and phenotype, we searched the database of UniProt and reported references and then summarized the reported mutation sites and clinical manifestations including childhood and adolescent cerebral ALD, adult ALD, AMN, adrenal insufficiency-only, asymptomatic or presymptomatic types, and complicated ALD which cannot be classified (Figure 5). X-ALD has high genetic heterogeneity. The clinical manifestations are highly variable even in the pedigree carrying same variant. The main phenotypes differ from different periods of the disease. The same variants can exhibit different clinical features. Thus, further effects may be needed to clarify the diversity in clinical manifestations and the correlationship of genotype-phenotype.

Therapeutic strategies include adrenocortical replacement; some of the adrenal insufficiency patients may require both glucocorticoid and mineralocorticoid to maintain water electrolyte balance. For severe adrenal insufficiency or even adrenal crisis, timely initiation of steroid replacement is essential. Nutrition intervention, for instance, low-fat diet and Lorenzo's oil may reduce VLCFAs level [36]. In this way, the risk of development with cerebral disorders may be reduced. However, they lack the evidence to improve the symptoms and progression. Another efficiency approach is hematopoietic stem cell transplantation (HSCT), especially in the early stage of the disease [37]. Transplantation with the autologous bone marrow transfected in vitro with *ABCD1* gene modification has been performed [38, 39]. Bone marrow transplant has been reported in CCALD therapy, and most of the patients achieved improved prognosis. However, the preexisted severe symptoms may influence the outcome after transplantation [40]. In animal experiments, Abcd1 mice show reduction of the mitochondrial biogenesis driven by the PGC-1*α*/PPAR*γ* pathway [41]. Oxidative stress is involved in the physiopathological mechanism of neurodegenerative cascade and mitochondrial depletion [42]. Pioglitazone, a PPAR*γ*/PGC-1*α* axis activator, may modulate the anti-inflammatory and antioxidant response. Recently, pioglitazone is under phase II clinical trials for adrenoleukodystrophy patients, revealing a potential for clinical translation [43]. Therapy options are limited; in addition, some therapeutic approaches relay on the early diagnosis and intervention. Therefore, gene sequencing analysis may be an important tool for newborn screening and genetic counseling.

## 4. Conclusions

In this study, we reported two novel mutations of *ABCD1* gene in men with X-ALD. These two cases were both considered as adrenal insufficiency at first and finally diagnosed with X-ALD when neurological manifestations occurred after several years. In these two pedigrees, we identified two novel mutations of *ABCD1* gene, NM_000033.3(ABCD1): c.874_876delGAG (p.Glu292del) and NM_000033.3(ABCD1): c.96_97delCT (p.Tyr33Profs^*∗*^161). The screening test for ALD, such as VLCFA concentration and brain MRI scan, should be considered in the early onset Addison's disease patients with the family history to screen X-ALD early.

## Figures and Tables

**Table 1:** Clinical characteristics and genetic analysis results of the patients.

No.	Sex	Family history	C24:C22	C26:C22	Cortisol (nmol/L)	ACTH (pg/mL)	Sodium (mmol/L)	Potassium (mmol/L)	Corticoid dosage	Mutation	Exon
Case 1	M	+	1.75	0.089	3.58	499.3	128	5.1	Hydrocortisone, 20 mg/d	c.874_876delGAG	1
Case 1	F	+	1.06	0.033	Nd	ND	138	4.8	None	c.874_876delGAG	1
Case 2	M	−	ND	ND	45.4	1071	125	5.3	Hydrocortisone, 30 mg/d	c.96_976delCT	1
Reference range			<1.39	<0.023	171-536	7.2-63.6	135-145	3.5-5.5	Fludrocortisone		

**Table 2:** Mutations of *ABCD1* gene in the pedigrees of X-ALD.

No.	Nucleotide mutations	Amino acid variants	Exon	Variation type	Inheritance	ESP6500	1000G	dbSNP	PROVEAN score	Mutation taster	Pathogenicity evaluation
Case 1	c.874_876delGAG	p.Glu292del	1	Deletion	Familial	ND	ND	ND	−13.987 (deleterious)	Disease causing	Likely pathogenic (PM2 + PM4, PP3 + PP4 + PP5)
Case 2	c.874_876delGAG	p.Tyr33Profs*∗*161	1	Deletion	Familial	ND	ND	ND	−6.7 (deleterious)	Disease causing	Pathogenic (PVS1, PM2 + PM4, PP3 + PP4 + PP5)

ND, not detected. Pathogenicity evaluation of American College of Medical Genetics (ACMG) guideline. PROVEAN score (cutoff = −2.5)

## Data Availability

The data used to support the findings of this study are included within the article.

## Abbreviations

X-ALD: X-linked adrenoleukodystrophy
ABCD1: ATP-binding cassette subfamily D member 1
ALDP: Adrenoleukodystrophy protein
VLCFAs: Very long-chain fatty acids
AMN: Adrenomyeloneuropathy
ACTH: Adrenocorticotropic hormone
MRI: Magnetic resonance imaging
ER: Endoplasmic reticulum
ROS: Reactive oxygen species.
